# Isolation Hospital Consultants' Fees

**Published:** 1922-04

**Authors:** Edwin H. T. Nash

**Affiliations:** Medical Officer of Health for the Heston and Isleworth U.D.C.


### CORRESPONDENCE.

[Correspondence on all subjects is invited, but we cannot in any way be responsible for the opinions expressed by our correspondents, who must give name and address as a guarantee of good faith, but not necessarily for publication. Correspondents are reminded that conciseness greatly facilitates early insertion.]

### Isolation Hospital Consultants' Fees.

*To the Editor of* The Hospital and Health Review.

Sir,—The paragraph in your March issue with regard to my opinion respecting the terms of office of the post of Consulting Physician to the local Isolation Hospital may possibly be taken to mean that the present Medical Superintendent has been in some way remiss in his duties. I wish to make it absolutely clear that there is not the slightest suggestion of such a thing. The present Medical Superintendent, I know, enjoys the complete confidence of his Committee.

In my opinion the idea of a fee per case in this matter is psychologically unsound. It is a question of general principle.

I am, yours faithfully,
Edwin H. T. Nash,
Medical Officer of Health for the Heston and Isleworth U.D.C.

### Edinburgh Royal Infirmary and Approved Societies.

*To the Editor of* The Hospital and Health Review.

